# Diagnosis patterns for rifampicin-resistant TB after onset of COVID-19

**DOI:** 10.5588/ijtld.21.0340

**Published:** 2021-09-01

**Authors:** E. Mohr-Holland, D. Hacking, J. Daniels, V. Scott, V. Mudaly, J. Furin, C. Pfaff, A. Reuter

**Affiliations:** 1 Médecins Sans Frontières (MSF), Khayelitsha, South Africa; 2 MSF Southern Africa Medical Unit, Cape Town, South Africa; 3 City of Cape Town Department of Health, Eastern Area, Cape Town, South Africa; 4 Provincial Government of the Western Cape, Cape Town, South Africa; 5 Harvard Medical School, Boston, MA, USA

Dear Editor,

SARS-CoV-2 has had a grave impact on preventive and curative TB services globally.^1^–^3^ In South Africa, there has been a 48% reduction in access to TB testing and a 33% reduction in the TB testing positivity rate since the initial outbreak of the epidemic.^4^,^5^ This is worrying, given the proven need for rapid diagnosis and treatment initiation to minimise the risks of TB transmission and ensuing morbidity and mortality.^6^ Additionally, TB, including rifampicin-resistant TB (RR-TB), is a risk factor for COVID-19 mortality.^7^ For more than a decade, Médecins Sans Frontières (MSF) has worked with the South African Department of Health (DoH) providing RR-TB services in Khayelitsha, South Africa, a setting with high burdens of TB/RR-TB and HIV.

In this study, we set out to describe how RR-TB diagnosis was impacted by the different waves of COVID-19, from its initial onset in March 2020 through to the recovery from the second wave in February 2021. We conducted a retrospective analysis of RR-TB diagnosis each quarter from January 2018 to March 2021 in Khayelitsha, South Africa. COVID-19 control measures and health care system adaptations were mapped to better understand how these might have impacted access to RR-TB diagnostic services during this period. Of note, Xpert® MTB/RIF Ultra (Cepheid, Sunnyvale, CA, USA) is the primary diagnostic test for TB in South Africa, and Ultra results provide data on the presence or absence of *Mycobacterium tuberculosis* and RR-TB. In response to COVID-19, the South African DoH (as advised by the ministerial advisory committee), implemented five levels of ‘lockdown’, with Level-1 the least restrictive and Level-5 the most restrictive.^8^ Level-5 included a complete ‘lockdown’ in which a person’s movements were restricted to buying food, providing essential services and accessing essential health services.

Overall, 628 patients were diagnosed with RR-TB over the study period. In 2020, 195 RR-TB patients were diagnosed, of which the majority were diagnosed in Q1 (35%, *n* = 69). Of the remaining patients, 17% (*n* = 33), 23% (*n* = 44) and 25% (*n* = 49) were diagnosed in respectively Q2, Q3, and Q4. There were marked decreases in monthly diagnosis, notably in April 2020 (see Figure and Table). In Q2 and Q3 of 2020, diagnosis declined by respectively 25% and 21% when compared to the same quarters in 2019 (33 vs. 42 and 44 vs. 56 cases, respectively); in Q4 of 2020, diagnosis was similar to that of Q4 in 2019 (49 vs. 46 cases). The Table shows the key COVID-19 control measures and resulting changes to the management of TB that might explain some of the trends in RR-TB diagnosis over this period. The initial decrease in RR-TB diagnosis corresponds to the re-orientation of primary health care (PHC) services to respond to COVID-19; this included repurposing some health care workers, particularly those working in TB services, to SARS-CoV-2 screening, triage and testing. PHC services were further disrupted during the first wave of COVID-19 when infections among PHC staff led to the subsequent closure of facilities. Additionally, the package of services offered was reduced to the provision of emergency services and dispensing of chronic medications only; all non-emergency services were provided without entry to the facilities or full consultations. The decrease in cases also coincides with the initial restrictive Level-5 ‘lockdown’ in which people were urged to ‘shelter at home’ unless in need of an essential service. Upon the shift to a less restrictive ‘lockdown’ in May 2020, the DoH recommended integrated TB/COVID-19 screening of PHC attendees. Moving into Q3, there was a slight increase in RR-TB diagnosis noted, which might also be related to the use of sputum for SARS-CoV-2 testing, which enabled easier integration of SARS-CoV-2 and TB testing. In August 2020, the ‘lockdown’ became even less restrictive and the provincial DoH released TB recovery plans, including an integrated TB/COVID-19 screening tool. Thereafter, in September 2020, the country moved to the least restrictive ‘lockdown’ level, with few restrictions on movement. However, diagnosis only started increasing in November, around which time the second wave of COVID-19 began. In Q1 2021, diagnosis again declined by 38% when compared to the same months in 2020 (43 vs. 69 cases).

**Figure:**
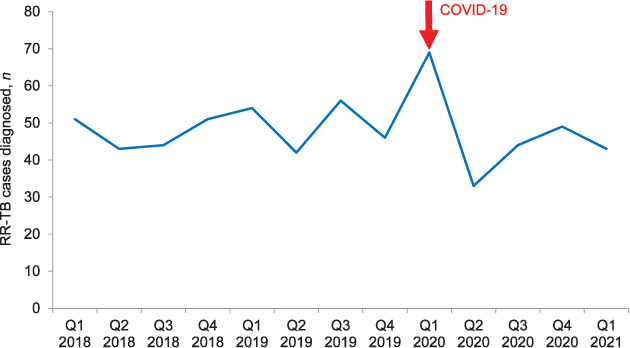
Number of RR-TB cases diagnosed per quarter, January 2018–March 2021, Khayelitsha, South Africa. RR-TB = rifampicin-resistant TB.

**Table:**
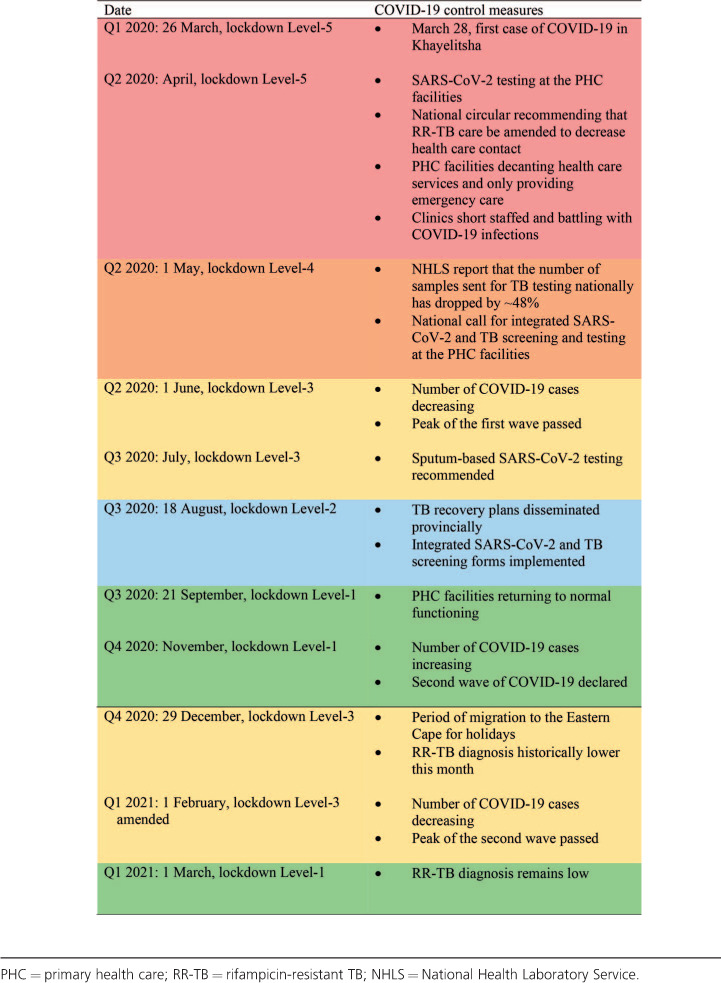
Timeline showing the implementation of lockdown levels, key COVID-19 control measures and corresponding changes to the management of TB in South Africa.

We found that RR-TB diagnosis decreased by 21–38% following the start of the pandemic. These findings are in line with the reductions in TB diagnoses reported both nationally^4^,^5^ and globally^2^,^3^,^9^–^11^ as a result of the multifaceted difficulties in accessing PHC services during this period.^12^ These challenges entailed limited access to PHCs due to restrictions on movement during higher levels of lockdowns (e.g., no public transportation, or fear of travel due to military presence enforcing lockdown measures), as well as the fear of contracting SARS-CoV-2 at the facilities. Furthermore, because of the overlap between TB and COVID-19 symptoms, patients who did present with TB symptoms and would thus normally have received TB testing, might instead have been screened and assessed as people under investigation for SARS-CoV-2 and only received a test for SARS-CoV-2. Finally, public messaging was initially focused on encouraging people to “stay at home when they are sick”; only later did the health minister reiterate that immunisations, TB and HIV services were functional and key. These challenges were observed globally and were more likely to have disproportionately affected individuals and communities impacted by TB.^13^ Our findings are even more worrying in the light of the recent South African prevalence survey results, which highlight that in 2018, 40% (*n* = 154,348) of people with TB in South Africa were never diagnosed, and thus did not receive treatment.^14^ This gap is likely to have significantly widened as a result of COVID-19. The Stop TB Partnership has estimated that there will be an additional 6.3 million TB cases between 2020 and 2025 as a result of a 3-month lockdown with a coinciding 10 month recovery period.^1^ Household transmission of TB/RR-TB might have increased due to the recommendations to ‘shelter at home’ that were detrimental to communities already plagued by poorly ventilated and overcrowded housing environments, which perpetuate TB transmission.^15^ It is worrying that despite a renewed political interest in implementing TB recovery plans, and integrated TB/SARS-CoV-2 screening protocols, TB diagnosis remains low.

These results are disturbing, and suggest that COVID-19 has had a sustained impact on the TB/RR-TB programme in Khayelitsha, which has not been fully addressed by integrated TB/SARS-CoV-2 screening, or the TB recovery plans. The reasons for this are multifactorial and there is a need for innovative thinking and rapid and concerted action in this area, particularly considering the ongoing third wave of COVID-19. A multipronged approach is needed to increase diagnostic testing including health promotion and community-based approaches, routine TB screening for all PHC users and household contact tracing for all newly diagnosed persons with TB.
